# Evaluation of Low-Cost GNSS Receiver under Demanding Conditions in RTK Network Mode

**DOI:** 10.3390/s21165552

**Published:** 2021-08-18

**Authors:** Daniel Janos, Przemysław Kuras

**Affiliations:** Information Technology in Civil Engineering Research Group, AGH University of Science and Technology, 30-059 Cracow, Poland; janos@agh.edu.pl

**Keywords:** low-cost GNSS receiver, u-blox ZED-F9P, network RTK, comparison, positioning accuracy, total station reference, survey-grade antenna

## Abstract

Positioning with low-cost GNSS (Global Navigation Satellite System) receivers is becoming increasingly popular in many engineering applications. In particular, dual-frequency receivers, which receive signals of all available satellite systems, offer great possibilities. The main objective of this research was to evaluate the accuracy of a position determination using low-cost receivers in different terrain conditions. The u-blox ZED-F9P receiver was used for testing, with the satellite signal supplied by both a dedicated u-blox ANN-MB-00 low-cost patch antenna and the Leica AS10 high-precision geodetic one. A professional Leica GS18T geodetic receiver was used to acquire reference satellite data. In addition, on the prepared test base, observations were made using the Leica MS50 precise total station, which provided higher accuracy and stability of measurement than satellite positioning. As a result, it was concluded that the ZED-F9P receiver equipped with a patch antenna is only suitable for precision measurements in conditions with high availability of open sky. However, the configuration of this receiver with a geodetic-grade antenna significantly improves the quality of results, beating even professional geodetic equipment. In most cases of the partially obscured horizon, a high precision positioning was obtained, making the ZED-F9P a valuable alternative to the high-end geodetic receivers in many applications.

## 1. Introduction

Geodetic measurement techniques are one of the basic solutions for monitoring displacements and deformations of buildings and ground surfaces [1,2]. They provide the representation of observation results in an external, absolute reference system. Among them, an important role is played by the GNSS (Global Navigation Satellite System) technology. Its contemporary applications have been reviewed in detail by Bock and Melgar [3]. In particular, for continuous monitoring real-time observations are especially important [4]. Achieving the highest possible accuracy is possible thanks to the development of effective error mitigation methods and noise modeling [5]. Thus, high-precision geodetic GNSS receivers are commonly used for construction sites that require high measurement accuracy. These solutions are often found for continuous monitoring of high buildings [6], bridges [7] or dams [8].

Various satellite positioning techniques developed over several decades have provided sufficient accuracy for many monitoring cases. However, in recent years there has been a significant progress in this field through the increasing availability of low-cost receivers. They are an alternative to geodetic-grade receivers and can be divided into two groups: low-cost receivers equipped with patch antennas and ultra-low-cost hardware, used e.g., in mobile phones [9,10]. These solutions significantly reduce the cost of observation in relation to precise geodetic equipment. They allow the use of a large number of measurement sensors on the surveyed object, especially in a situation where, due to the possible destruction of the monitored object, they should be treated as “throwaway instrumentation” [11]. Low-cost equipment has been found to be sufficiently accurate for some applications in geodetic monitoring of natural hazards [12]. The suitability of these devices for monitoring over short baselines at accuracy levels better than 3 mm has also been confirmed by Caldera et al. [13]. On the other hand, studies performed by Garrido-Carretero et al. [14] demonstrated the accuracy of the RTK solution using low-cost receivers on the level of 5.5 mm and 11 mm for the horizontal (Hz) and vertical (V) components, respectively. This result turned out to be 2–3 times worse compared to geodetic-grade equipment. Another application is the determination of positions of moving vehicles, especially with the additional use of inertial sensors [15,16]. Low-cost receivers have been also included in various applications such as landslide monitoring [17] or detection of water vapor time variations [18]. All in all, the implementation of such devices should be preceded by studies in the field of positioning accuracy.

Since low-cost GNSS receivers, and especially dual-frequency ones, have been available for a short time, the number of studies on their accuracy is quite small. Since their appearance on the market, they have become the subject of research, both in terms of determining the position using the static [19] and kinematic [20] methods. Comprehensive research of low-cost single-frequency receivers was performed by Jackson et al. [21]. Three of the five receivers achieved horizontal position accuracies better than 2.6 cm in RTK mode, under favorable observing conditions. However, the authors found significant limitations on an accurate position using single-frequency receivers for dynamic applications.

Three years ago, the ZED-F9P low-cost dual-frequency GNSS receiver, manufactured by the Swiss company u-blox, appeared on the market. As a standard it is equipped with an ANN-MB-00 patch antenna with a ground plane. It provides signal tracking for all of the GNSS systems (GPS, GLONASS, Galileo and BeiDou). The accuracy of the RTK receiver is specified at 1 cm + 1 ppm CEP (Circular Error Probable) for horizontal and vertical positions. This value is independent of the number of satellite navigation systems used, i.e., it is the same for both 4 systems (GPS + GLO + GAL + BDS) and one (GPS) [22]. This receiver, offering wide usability, has become the subject of research in the field of e.g., positioning accuracy.

Wielgocka et al. [23] tested the ZED-F9P receiver for positioning accuracy using different methods. In static mode, they obtained RMSE (root mean square error) values of 11, 17 and 15 mm for north, east and up components, respectively, based on differences from reference data. In contrast, in RTK mode, they found that the manufacturer’s parameters were not met and were 20 mm and 53 mm (both RMSE) for horizontal and vertical components, respectively, for a baseline shorter than 0.5 km. In addition, it was noted that the main source of height error was the use of low-cost antennas. Further tests for this receiver were performed by Hamza et al. [24], who used low-cost antennas from various manufacturers to determine position using the static method. Their results allowed them to conclude that low-cost instruments give a coordinate accuracy of a few millimeters, but their precision is four times worse than that of geodetic receivers (based on adjustment of the established geodetic network). A detailed study on the influence of a patch antenna was carried out by Krietemeyer et al. [25] by comparing it with geodetic-grade antennas, with and without consideration of antenna-relative calibrations.

A critical component of the low-cost receivers is the patch antenna, due to the noticeable degradation in positioning accuracy. For this reason, they have been tested using high-precision geodetic antennas. Thus, Tsakiri et al. [26] verified the accuracy of single-frequency u-blox receivers. The results obtained were comparable to geodetic receivers—not exceeding 0.005 m (2σ) in all components (for a baseline shorter than 0.5 km) and 0.02 m (for up to 18 km long baseline). Even better results were obtained by Poluzzi et al. [27], obtaining RMSEs below 2 mm and about 5 mm for the horizontal (Hz) and vertical (V) components, respectively, based on 1 h static observations. Moreover, they estimated the accuracy of the real-time solution as 4 mm (Hz) and 8 mm (V) RMSE. A similar RTK mode study was performed by Semler et al. [28], comparing the ZED-F9P results with a professional geodetic receiver. They obtained a 3D position standard deviation value of 7 mm, which they considered excellent for low-cost GNSS equipment compared to a value of 13 mm obtained with a high-end receiver.

It is worth noting that the tests mentioned above were usually conducted under favorable field conditions, i.e., with a large number of available satellites and an open horizon. However, terrain obstacles are a significant handicap in many practical cases, so it seems reasonable to perform tests under such conditions. The tests carried out in this thesis take into account different terrain situations, ranging from an open-sky environment, through a horizon obscured from different directions, to the case of an urban canyon.

Furthermore, in testing the accuracy of the devices, it is crucial to provide reliable reference values. Most comparisons utilize results obtained with different GNSS receivers, equipped with various antennas (patch and geodetic), using other positioning methods. In this paper, satellite observations are compared with total station measurements, which provide higher accuracy and are less dependent on random factors such as satellite availability or multipath effect.

The motivation for undertaking the research was to verify the accuracy of a low-cost dual-frequency GNSS receiver operating in RTK mode in relation to high-precision observations. An essential element of our work was to create diverse field conditions in which low-cost receivers can be used, such as during vehicle positioning.

Furthermore, we conducted tests by introducing a mid-cost solution, expecting worse results for low-cost receivers. A high-precision geodetic antenna was combined with a low-cost receiver. Despite the higher cost of the antenna, this combination is still a cost-effective alternative to high-end geodetic equipment in situations where high positioning accuracy is required.

A complementary objective of our work was to provide further research results on the accuracy of low-cost dual-frequency GNSS receivers to the relatively small base available in the scientific literature. The large number of tests performed in different conditions and locations worldwide will contribute to a statistically reliable evaluation of equipment with high potential applications.

## 2. Materials and Methods

### 2.1. Low-Cost U-Blox Global Navigation Satellite System (GNSS) Receiver

The C099-F9P application board has been used to test the u-blox ZED-F9P GNSS receiver. It is a two-frequency, four-system receiver—it receives GPS (L1C/A, L2C), GLONASS (L1OF, L2OF), Galileo (E1B/C, E5b) and BeiDou (B1l, B2l) signals [22]. It offers RTK and RTN operation with high frequency (up to 20 Hz) and accuracy (±1 cm + 1 ppm). In conditions of good satellite visibility, the receiver quickly resolves its position (cold start < 24 s, reacquisition < 2 s). Also, anti-jamming and anti-spoofing algorithms are implemented into the receiver, allowing the assumption that it can discard unwanted signals. It has a wide operating temperature range, low power consumption, light weight and a large number of physical inputs/outputs and communication capabilities. The parameters of this device declared by the manufacturer, its price, as well as programming libraries available on the Internet, provide great opportunities for testing this receiver.

### 2.2. Professional Land Surveying GNSS Receiver

At the same time, measurements were taken with a professional geodetic GNSS receiver of a proven manufacturer, Leica GS18T [29], to compare the quality of the satellite measurements. It also has advanced technologies, such as the ability to compensate for pole tilts, or advanced measurement applications, while the following tests will take into account the performance only of the satellite measurement.

Unlike the C099-F9P, the Leica GS18T receiver forms a single unit with the antenna —a “smart antenna” device. This one has its advantages and disadvantages, such as not being able to freely change the antenna itself or the high weight of the whole. This also causes the u-blox to have an advantage in the flexibility of mounting the device (for example, on autonomous cars, drones, machines or buildings).

### 2.3. GNSS Antennas

Since the C099-F9P is an application board with the receiver itself, an active GNSS antenna must be connected to it. The manufacturer includes a panel antenna (model ANN-MB-00), which should provide the required accuracy in conditions with good visibility of satellites. It is small (its dimensions are only 60.0 × 82.0 × 22.5 mm) and weatherproof (protection level IP67).

**Figure 1:**
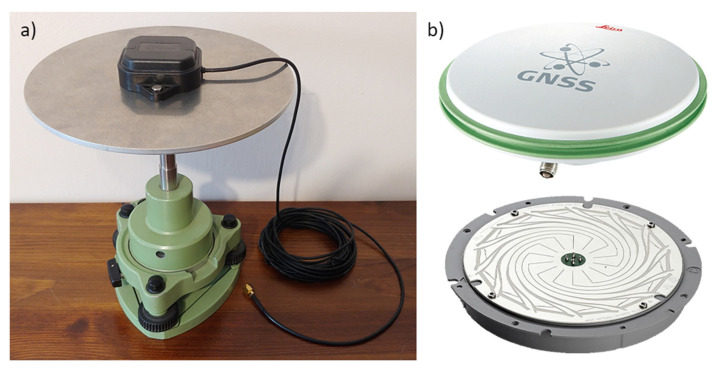
Antennas used in conducted experiment: (**a**) u-blox ANN-MB-00 antenna with self-made aluminum ground plane; (**b**) survey-grade antenna Leica AS10 outside and inside (NovAtel Pinwheel technology) view.

To reduce the number of reflections from other objects and/or the environment reaching the antenna, when mounting the above antenna, it should be ensured that it is placed on a plate made of a conductive metal (so-called ground plane). For the following tests, a special 4 mm thick disc with a diameter of 200 mm and a bracket for it were self-made of aluminum. The assembly is shown in Figure 1a. The whole set provides a coaxial mounting with the second antenna and the Leica GS18T receiver, allowing straightforward interpretation of the results later in the tests.

To check the performance of the ANN-MB-00 antenna in multipath reduction, measurements were additionally performed with a professional surveying antenna Leica AS10, which was applied in another similar test [14,30]. This uses, among other things, NovAtel’s advanced Pinwheel technology [31] (Figure 1b), which, as will be shown later, helped to obtain better results.

To achieve the best possible stability of the antennas during the survey, geodetic tripods were used together with Leica tribrachs and antenna carriers.

During precise geodetic measurements, PCO (Phase Center Offset) should also be taken into account. According to the manufacturer’s specifications [32], the PCO of the u-blox ANN-MB-00 antenna is 8.9 mm and 7.6 mm in the vertical (V) plane for L1 and L2 signals, respectively, and less than 5 mm in the horizontal (Hz) plane for both frequencies. For the Leica AS10 antenna, on the other hand, the values are 58.3 mm and 55.5 mm for L1 and L2 signals (V) and less than 3 mm (Hz) for both frequencies [33].

Unfortunately, it is not possible to upload an antenna calibration file to the ZED-F9P receiver. Due to the accuracy of the RTK measurement and the small PCO values in the horizontal plane, they were omitted. However, in the vertical plane, these values were included. Because the differences between the PCO for L1 and L2 are less than 3 mm, and the PCVs (phase center variations) are of the order of 5 and 10 mm for L1 and L2 [32], the PCO values for the L2 signal were used in post-processing.

### 2.4. Experiment Setup and Reference Data

**Figure 2:**
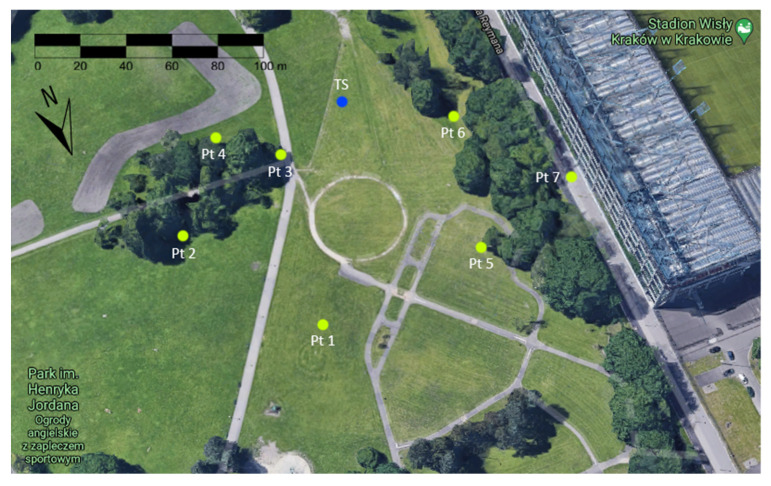
Sketch of measurement site on Google Maps 3D.

Measurement stations were planned at locations with different horizon exposure conditions, so that the test would be reliable and different results could be obtained. A sketch of the measured points (Figure 2) shows approximately the actual conditions in the field. Measurements were made in May, when trees already start to gather leaves and constitute a barrier for a satellite signal. Results of these observations are presented in Table 1. Station 1 had a perfect exposure of the horizon, stations 2–4 had the horizon covered only from one side, station 5 had in addition tree branches directly above it, station 6 was surrounded by trees and a nearby hill, while station 7 was located in an “urban canyon”.

### 2.5. Measurements Method

The measurements with both receivers—Leica GS18T and u-blox ZED-F9P—were performed in RTK Network mode. In this mode Leica declares a slightly higher measurement accuracy of its receiver due to the distance to the base [29]. To obtain consistent results, in both cases, RTCM corrections to the observations were taken from the same network (Leica Smart Net Poland), source NAVGEO_VRS_3_2 (GPS + GLO + GAL + BDS).

**Figure 3:**
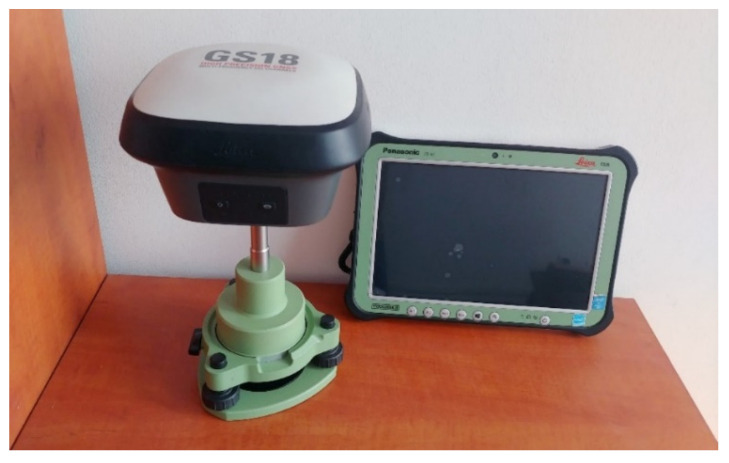
Leica GS18T receiver with Leica CS35 controller.

**Figure 4:**
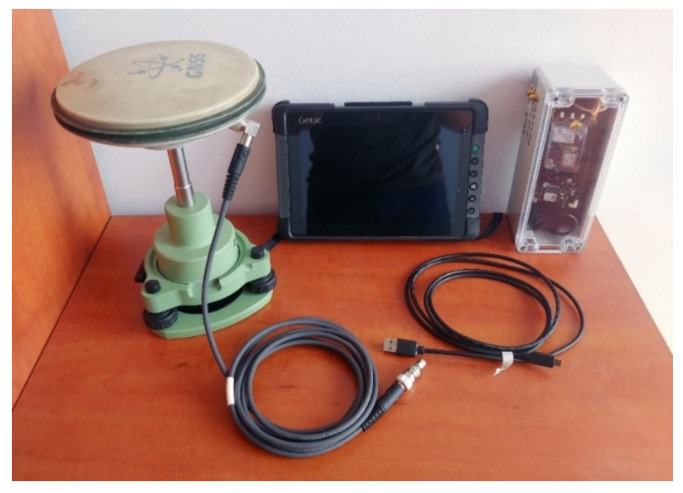
U-blox C099-F9P application board in self-made case with powerbank, Getac T800G2 rugged tablet with custom survey software, Leica AS10 antenna and cables.

The measurement at the station consisted of at least five measurement series (the exception was station 7) for each hardware configuration. Measurements in successive configurations (Leica GS18T (Figure 3), u-blox ZED-F9P + Leica AS10 (Figure 4), u-blox ZED-F9P + ANN-MB-00 (Figure 1a and Figure 4)) were performed alternately to ensure that each receiver had the most similar measurement conditions (access to the same satellites).

The scheme of one measurement series on the station was as follows:

- receiver initialization—maximum 30 s,
- measurement (collection of observations) —30 s,
- change of antenna/receiver.

The collected observations in one 30-s measurement were averaged. After completing the measurement at the station, at least five separate (averaged) measurements of each antenna were obtained and taken for further analysis.

### 2.6. Accuracy Assessment

**Figure 5:**
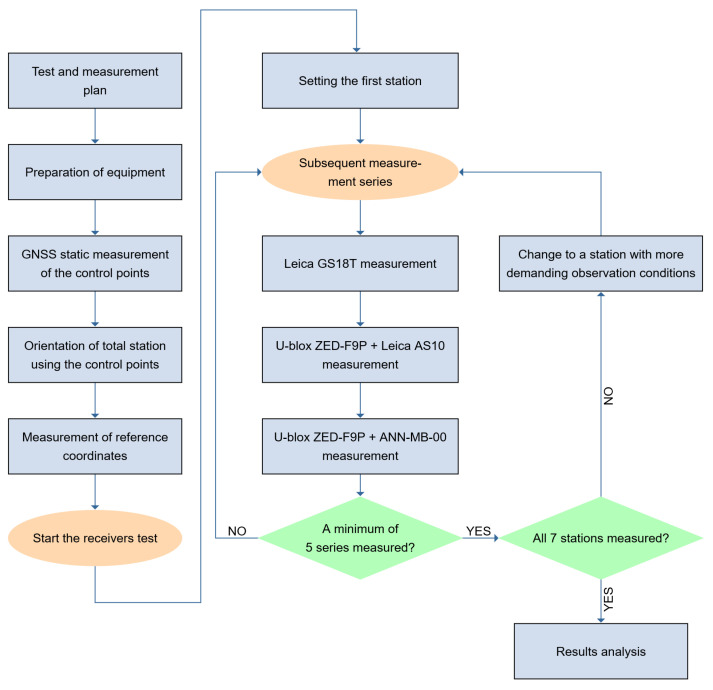
Block-chart of the experiment.

The block-chart of all tests and measurements is presented in Figure 5.

The collected observations in one 30-s measurement series were averaged (Equation (1)). After measuring with each antenna in at least 5 series (Equation (2)), the results were taken for further analysis. The differences from the reference coordinates (Equation (3)) and standard deviations of the measured coordinates (Equation (4)) were calculated for each station and each hardware configuration.

(1) $$OMS=\frac{\sum_{i=1}^{n=30s}\;a_{i}}{n}$$

where:

OMS—one measurement series;
n—number of sub-measurements (observations) until the time reaches 30 s;
$a_{i}$—sub-measurement (observation) of X, Y and H coordinates.

(2) $$MMS=\frac{\sum_{i=1}^{m}OMS_{i}}{m}$$

where:

MMS—mean measurement series;
m—number of measurement series (mostly 5).

(3) $$RD=a_{R}-MMS$$

where:

RD—difference between reference and mean measured coordinates (Ref.);
$a_{R}$—reference X, Y or H coordinate.

(4) $$SD=\sqrt{\frac{\sum_{i=1}^{m}{\left|OMS_{i}-MMS\right|}^{2}}{m}}$$

where:

SD—standard deviation of X, Y or H coordinate (St. deviation);
m—number of measurement series (mostly 5).

## 3. Results

### 3.1. Open-Sky Conditions

The test of the ZED-F9P receiver started with measurements under good satellite visibility conditions. The Pt 1 measurement station had no significant obstacles around it. Table 2 contains results for different hardware configurations. The columns contain the averaged coordinates from the five measurement series (MMS), the differences of these coordinates with respect to the reference ones (RD) and their standard deviations, calculated based on the five measurement series (SD). 

**Figure 6:**
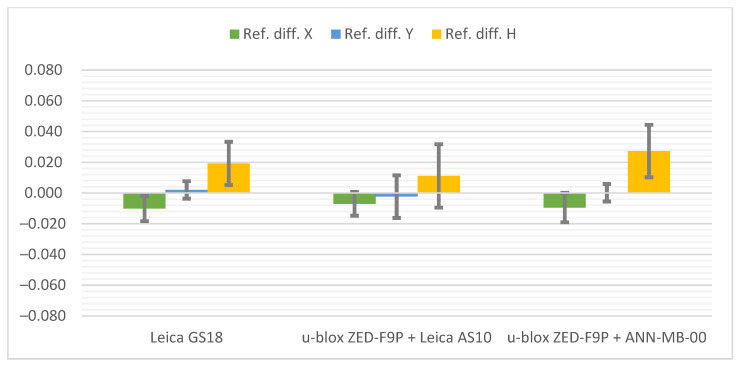
Cumulative graphs of measurement results at the Pt 1 station (open sky conditions; differences in relation to the reference points with standard deviations [m]).

Below (Figure 6) there are cumulative graphs, clearly illustrating the results obtained. There are differences from the reference coordinates with standard deviation bars on it. Both receivers meet the requirements in terms of general RTK Network measurement accuracy. It is worth mentioning that the most convergent coordinates to the reference ones were obtained with the u-blox ZED-F9P + Leica AS10 antenna set. On the other hand, the standard deviations present the slightly higher repeatability of the Leica GS18T. The increase in measurement accuracy in the vertical plane of the ZED-F9P receiver after replacing the antenna with the Leica AS10 can also be seen.

### 3.2. Nearly Open-Sky Conditions

In the next stage of the tests, measurement stations were located in places with one side of the horizon obscured. Those sides were the southern, eastern, and northern for Pt 2, Pt 3 and Pt 4 stations, respectively. Tall trees limited the horizon. The results for each station, in turn, are presented below (Table 3, Table 4 and Table 5).

**Figure 7:**
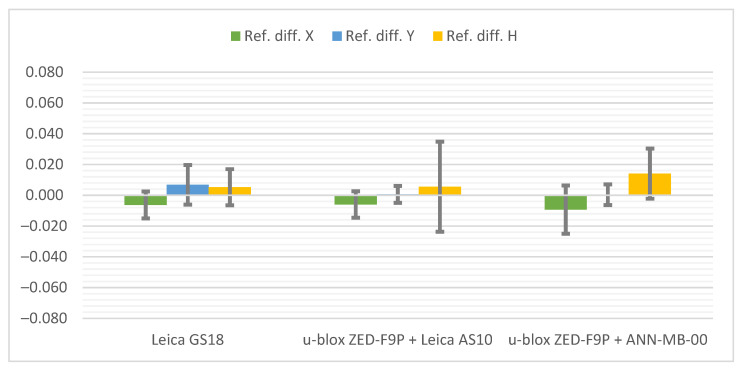
Cumulative diagrams with results of measurements at the Pt 2 station (horizon obscuration from the south; differences in relation to the reference points with standard deviations [m]).

**Figure 8:**
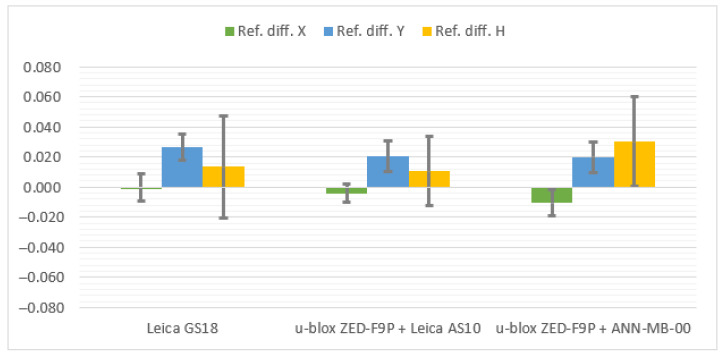
Cumulative diagrams with results of measurements at the Pt 3 station (obscuring the horizon from the east; differences in relation to the reference points with standard deviations [m]).

**Figure 9:**
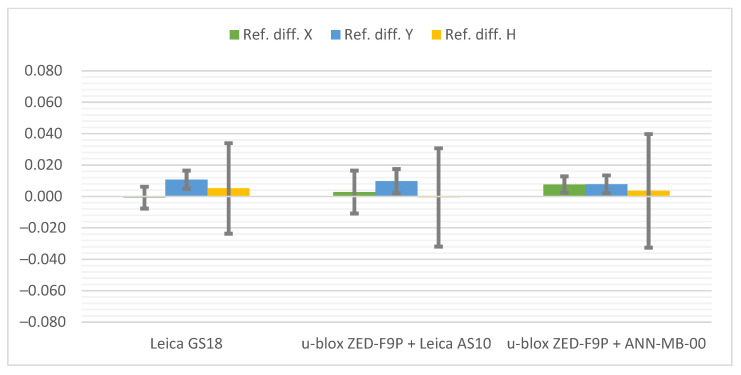
Cumulated diagrams with results of measurements at the Pt 4 station (obscured horizon from the north; differences in relation to the reference points with standard deviations [m]).

Below (Figure 7, Figure 8 and Figure 9) the cumulative graphs showing the results are presented. It can be noticed that the horizon obscuration from one side adversely influenced the values of standard deviations, especially of the vertical component. The eastern horizon obscuration at station Pt 3 influenced the Y coordinate error (running along the west–east direction). 

The results on all stations are satisfactory, although the Leica GS18T stays slightly behind the u-blox ZED-F9P + Leica AS10 antenna set in terms of differences to reference coordinates. Once again, there is also an increase in positioning accuracy favoring the Leica AS10 antenna over the u-blox ANN-MB-00.

### 3.3. Forest and Mountain-Like Conditions

The subsequent measurement stations were located in even more demanding conditions. Point Pt 5 was planned with the horizon obscured from the west (by trees and a nearby football stadium). Additionally, the tripod was set up directly under the tree branches. This was expected to degrade the accuracy of the position measurement significantly. The Pt 6 station, on the other hand, was located in the surroundings of less dense trees, however, on all sides. Additionally, on the southern side the horizon was limited by a nearby mound (sledge hill) and on the western side by the football stadium, as mentioned earlier. The results for each station are presented below (Table 6 and Table 7).

**Figure 10:**
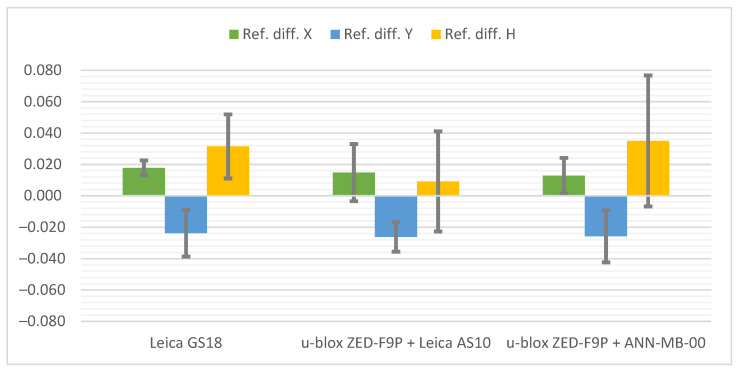
Cumulated diagrams with results of measurements at the Pt 5 station (obscured horizon from the west and the top; differences in relation to the reference points with standard deviations [m]).

Below (Figure 10) the cumulative graphs for the Pt 5 station are presented, where a significant deterioration in accuracy can be noticed. Because of the horizon obscuration from the west, the biggest differences in relation to the reference coordinates are again visible on the Y axis. It is worth noting that one of the measurements with the u-blox ZED-F9P + ANN-MB-00 set had a float type solution. The results presented here represent the other 4 measurements with a fixed type solution. Therefore, once again it can be seen that the difference of antennas is in favor of a professional GNSS antenna. Nevertheless, in the tested conditions all hardware configurations performed similarly, with an accuracy of ±3 cm. The smallest differences from the reference coordinates were achieved by the u-blox ZED-F9P + Leica AS10 set, while the highest measurement repeatability and the lowest standard deviation was achieved by the Leica GS18T.

**Figure 11:**
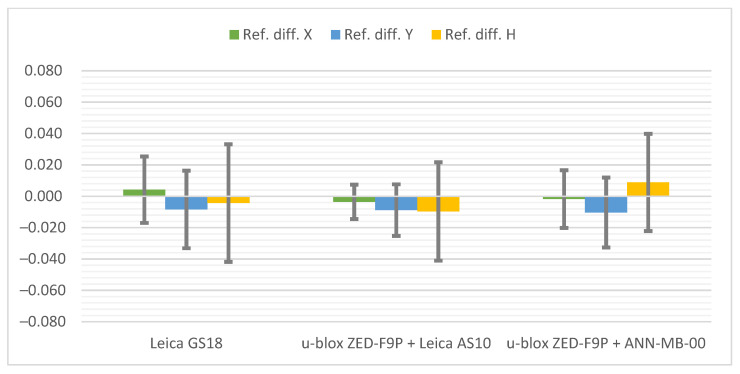
Cumulated diagrams with results of measurements at the Pt 6 station (obscuring the horizon from many directions; differences in relation to the reference points with standard deviations [m]).

At the Pt 6 station, results on the differences (Figure 11) are surprisingly good, within ±1 cm, and therefore similar to the Pt 1 station with a fully exposed horizon. The influence of the environment can only be seen in the bars of standard deviations. The smallest differences from the reference coordinates were obtained by the Leica GS18T receiver, slightly ahead of its rival, while the values of standard deviations definitely favor the u-blox ZED-F9P + Leica AS10 antenna kit.

### 3.4. Urban Canyon Conditions

**Figure 12:**
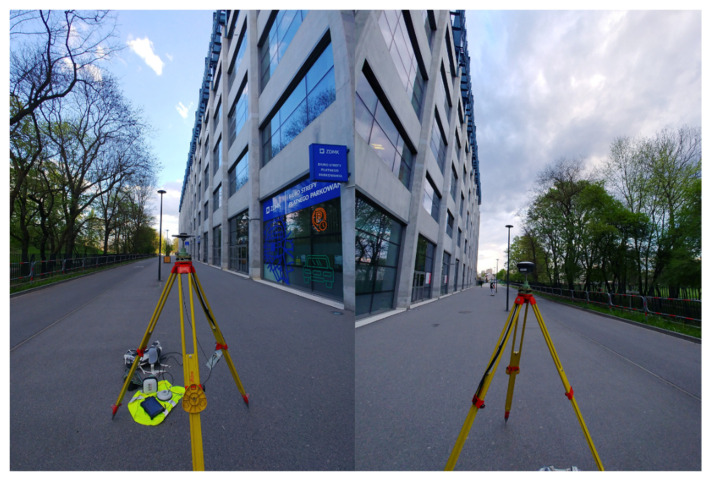
Field conditions at the Pt 7 station.

The last station was located in difficult measuring conditions for GNSS technology (Figure 12). On the west side the horizon was directly limited by a building, while on the east side by a line of tall trees—the tripod was set up along an alley running between a park and a large football stadium.

It is worth mentioning the problems that were encountered during the measurements. As many as eight series of measurements were performed on this station. The Leica GS18T receiver had a fixed solution only in half of the series. The ZED-F9P receiver with the Leica AS10 antenna had even fewer fixed solutions—only three. The same receiver, but with the ANN-MB-00 antenna, had fixed solutions in four series. Unfortunately, the result of one of them differed considerably from the reference coordinates—by as much as 13 m on the vertical coordinate. This measurement was removed from further considerations, nevertheless the result is still not satisfactory. The measurement results (values only from measurements with a fixed solution) are presented below (Table 8).

**Figure 13:**
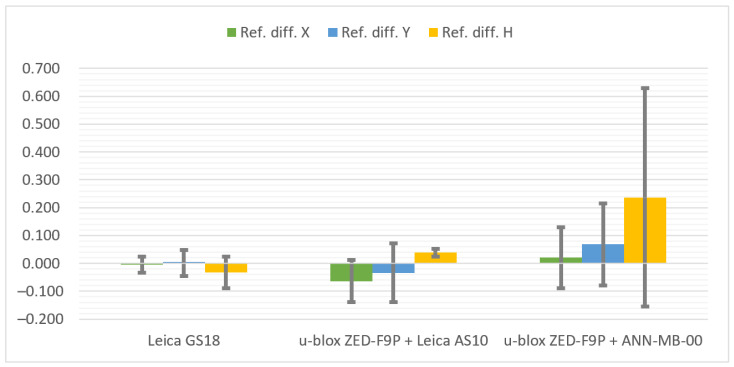
Cumulated diagrams with results of measurements at the Pt 7 station (urban canyon conditions; differences in relation to the reference points with standard deviations [m]).

The cumulative graphs representing the measurement results at the Pt 7 station are presented below (Figure 13). It is worth noting that the scale of the vertical axis has been changed from the previous graphs due to the large values of coordinate differences and standard deviations when measuring with the ANN-MB-00 antenna.

The best result was finally achieved by the Leica GS18T. As it is a professional geodetic receiver, its advantage over the low-cost u-blox ZED-F9P was expected. Nevertheless, it had not been apparent until the measurement on this station. Previously, the Leica GS18T has achieved even slightly worse results than the u-blox ZED-F9P configuration with the Leica AS10 antenna. However, in the last station the measurement was not problem-free—the receiver had 4 fixed position solutions for 8 measurements done. On the other hand, when the measurement was already taken with such a solution, then the appropriate accuracy was kept (in contrast to one measurement by ZED-F9P with ANN-MB-00 antenna) and the differences were on a similar level to the stations presented before. The conditions influenced noticeably also the standard deviations.

The configuration of the u-blox ZED-F9P + Leica AS10 antenna coped reasonably well. The problems that the receiver had in these conditions can be seen both in the type of the solution and the differences in relation to the reference coordinates, as well as in the values of the standard deviations.

The ANN-MB-00 antenna definitely did not cope with the signals reflected from the surrounding building and trees, which turned into a significant loss of accuracy, especially in the vertical plane. An additional disappointment was the solution marked as the fixed one while the difference between the reference coordinates exceeded 13 m.

## 4. Discussion

### 4.1. Sum of Absolute Coordinates Differences

**Figure 14:**
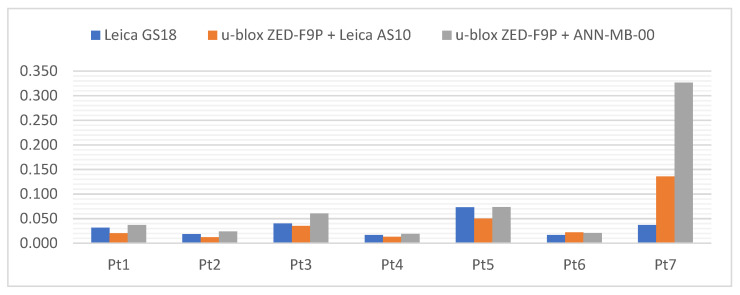
Sum of X, Y and H absolute differences [m] on each station point.

Figure 14 illustrates the summed coordinate differences, which were calculated using the formula:

(5) $$sum_{XYH}=\left|d_{X}\right|+\left|d_{Y}\right|+\left|d_{H}\right|$$

where:

$d_{X},\;d_{Y},\;d_{H}$—differences from the reference coordinates.

The Leica GS18T obtained the smallest sum (the highest measurement accuracy) at the Pt 4 station (horizon covered from the north) and it was 1.7 cm. On the other hand, the highest sum (the worst measurement accuracy) at the Pt 5 station (horizon covered from the west and directly from above) was 7.3 cm.

The u-blox ZED-F9P with the Leica AS10 professional surveying antenna had the smallest sum at the Pt 2 station (horizon obscured from the south) and it was 1.2 cm, while the largest sum at the Pt 7 station, with a value of 13.6 cm.

The u-blox ZED-F9P set with the standard ANN-MB-00 antenna attached by the manufacturer to the C099-F9P application board obtained the smallest sum at the Pt 4 position and it was 1.9 cm, while the highest sum at the Pt 7 position, with the value of 32.6 cm.

An increasing trend of results can be seen in the graph. Surprisingly, better results were obtained at station Pt 6 than at the station Pt 1 with a fully open horizon. It can be assumed that despite many obstacles on the horizon from different sides, there were also free gaps that made it possible to create a good-quality angular resection of signals coming from the satellites of all constellations.

Considering the conditions at all measurement stations, it can be concluded that both receivers performed well. The u-blox ZED-F9P, equipped with the Leica AS10 antenna, had a slight advantage in the clear areas, while its rival excelled in the very demanding “urban canyon” type conditions at the last station.

### 4.2. Mean Standard Deviations

**Figure 15:**
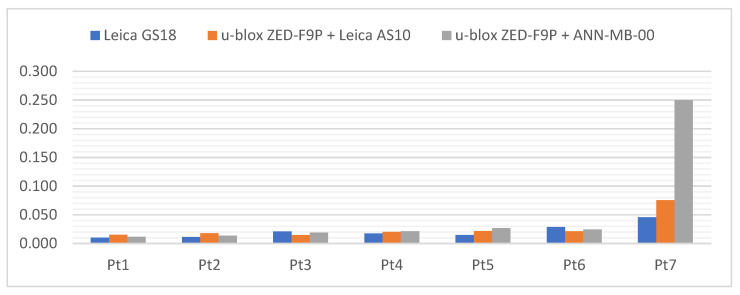
The MSD values [m] on each station point.

The last graph (Figure 15) shows the mean standard deviations. Such an indicator was used to express in the best way the average quality and reliability of the measurement with a given GNSS receiver. This indicator is calculated from the formula:

(6) $$MSD\;=\;\sqrt{\frac{{\sigma_{X}}^{2}+{\sigma_{Y}}^{2}+{\sigma_{H}}^{2}}{3}}$$

where:

$\sigma_{X},\;\sigma_{Y},\;\sigma_{H}$—standard deviations of the determined coordinates.

All receivers had the smallest MSD value at Pt 1 and the highest at Pt 7. These values were 1.0 cm and 4.6 cm for Leica GS18T, 1.5 cm and 7.5 cm for u-blox ZED-F9P + Leica AS10, and 1.1 cm and 25.0 cm for u-blox ZED-F9P + ANN-MB-00, respectively.

On the chart below (Figure 15) the increasing tendency of the MSD values can be clearly seen. This is a result of measurement difficulties at subsequent stations (more and more obscured horizon, access to less satellites of a given constellation, as well as more reflected signals and interferences). This proves a good choice of successive measurement stations in order to increase the difficulty of measurement.

## 5. Conclusions

The u-blox ZED-F9P performs very well for a GNSS receiver in this price range. The ANN-MB-00 antenna is suitable for precise measurements and provides centimeter accuracy, but only in conditions with sufficient horizon exposure. The ZED-F9P configuration with the ANN-MB-00 antenna provides an excellent relation of positioning quality to price, as well as size, weight or power consumption. This makes the u-blox a very versatile receiver that can be used in many industries, such as autonomous vehicles, building monitoring, surveying, robotics and marine. The simple configuration of the u-blox C099-F9P application board and the management of the ZED-F9P via the u-center software supplied by the manufacturer provides the user with an accurate position just minutes after unpacking the device for the first time. Software libraries available on the Internet further assist in implementing the device in individual research and projects. Additionally, the manufacturer provides user support through reliable, comprehensive technical documentation, and many solved problems can also be found in online forums as the u-blox ZED-F9P becomes increasingly popular.

In the case of positioning in more challenging conditions and/or with greater precision, a replacement for the ANN-MB-00 antenna with a model from another manufacturer of the survey-grade types would be worth considering. The Leica AS10 antenna has significantly improved the measurement performance of the u-blox ZED-F9P, with smaller PCVs (phase center variations) and more efficient multipath reduction. The u-blox ZED-F9P configuration with the Leica AS10 antenna turned out to be worse than the Leica GS18T professional geodetic receiver only in the last position, with better results on the previous positions. It can be assumed to be a promising outcome, considering the price gap between both devices.

The u-blox receiver was the most disappointing on the last station—not because it could not measure as accurately as the Leica GS18T, but because it indicated a fixed solution that differed very much from the reference coordinates. This fact causes uncertainty when measuring with this receiver in conditions of insufficiently good visibility and satellite configuration or placement. The Leica GS18T, unlike the u-blox, either maintained accuracy and marked the measurement as fixed, or the solution status changed to float, and the user had no doubts about the quality of the measurement. The manufacturer of the ZED-F9P should apply additional filters that would more accurately help to remove the multipath effect. In addition, measurement confidence should be increased or the insufficient measurement confidence should be alerted.

Reference coordinates were determined with a precision of one order of magnitude higher than the one of RTK/RTN measurements. Measurement series were performed alternately to ensure that the satellite configuration was as similar as possible between measurements in successive hardware configurations. The measurement stations were placed in locations with increasingly difficult measurement conditions, as shown in Figure 15 by the increasing coordinate differences and variations in standard deviations. Therefore, it can be concluded that the tests were carried out reliably, as shown in the results above.

In the subsequent tests, it is planned to check the positioning quality of the u-blox ZED-F9P receiver on the move to recognize even better its applicability in the automotive industry. Based on the measurement conditions of the above test, a suitable route will be proposed, as well as an appropriate quality reference positioning (robotic total station with the 360-degree prism, coaxially mounted with the ANN-MB-00 antenna, through a unique adapter made by 3D printing technology).

## Figures and Tables

**Table 1:** Reference points coordinates with position and height mean square errors.

Point	X [m]	Y [m]	H [m]	σ_2D_ [m]	σ_H_ [m]
Pt 1	5,548,070.0847	7,422,309.5843	204.0463	0.0020	0.0020
Pt 2	5,548,005.0782	7,422,333.4061	204.0551	0.0024	0.0026
Pt 3	5,547,998.8791	7,422,277.4878	204.3317	0.0023	0.0024
Pt 4	5,547,978.1491	7,422,301.2729	203.9264	0.0023	0.0025
Pt 5	5,548,077.6307	7,422,240.0247	204.0367	0.0024	0.0025
Pt 6	5,548,031.7399	7,422,213.1859	204.5718	0.0023	0.0025
Pt 7	5,548,075.2796	7,422,190.9203	205.9131	0.0024	0.0026

**Table 2:** Measurement results on Pt 1.

Receiver		Coordinate	Ref. Difference	St. Deviation
	X [m]	5,548,070.095	−0.010	0.008
GS18T	Y [m]	7,422,309.582	0.002	0.006
	H [m]	204.027	0.019	0.014
	X [m]	5,548,070.092	−0.007	0.008
ZED-F9P + AS10	Y [m]	7,422,309.587	−0.002	0.014
	H [m]	204.035	0.011	0.021
	X [m]	5,548,070.094	−0.009	0.010
ZED-F9P + ANN-MB-00	Y [m]	7,422,309.584	0.000	0.006
	H [m]	204.019	0.027	0.017

**Table 3:** Measurement results on Pt 2.

Receiver		Coordinate	Ref. Difference	St. Deviation
	X [m]	5,548,005.085	−0.006	0.009
GS18T	Y [m]	7,422,333.399	0.007	0.013
	H [m]	204.050	0.005	0.012
	X [m]	5,548,005.084	−0.006	0.009
ZED-F9P + AS10	Y [m]	7,422,333.406	0.001	0.006
	H [m]	204.050	0.006	0.029
	X [m]	5,548,005.088	−0.009	0.016
ZED-F9P + ANN-MB-00	Y [m]	7,422,333.406	0.000	0.007
	H [m]	204.041	0.014	0.016

**Table 4:** Measurement results on Pt 3.

Receiver		Coordinate	Ref. Difference	St. Deviation
	X [m]	5,547,998.879	0.000	0.009
GS18T	Y [m]	7,422,277.461	0.027	0.009
	H [m]	204.318	0.014	0.034
	X [m]	5,547,998.883	−0.004	0.006
ZED-F9P + AS10	Y [m]	7,422,277.467	0.020	0.010
	H [m]	204.321	0.011	0.023
	X [m]	5,547,998.890	−0.011	0.009
ZED-F9P + ANN-MB-00	Y [m]	7,422,277.468	0.020	0.010
	H [m]	204.301	0.030	0.030

**Table 5:** Measurement results on Pt 4.

Receiver		Coordinate	Ref. Difference	St. Deviation
	X [m]	5,547,978.150	−0.001	0.007
GS18T	Y [m]	7,422,301.262	0.011	0.006
	H [m]	203.921	0.005	0.029
	X [m]	5,547,978.146	0.003	0.014
ZED-F9P + AS10	Y [m]	7,422,301.263	0.010	0.008
	H [m]	203.927	−0.001	0.031
	X [m]	5,547,978.142	0.008	0.005
ZED-F9P + ANN-MB-00	Y [m]	7,422,301.265	0.008	0.006
	H [m]	203.923	0.004	0.036

**Table 6:** Measurement results on Pt 5.

Receiver		Coordinate	Ref. Difference	St. Deviation
	X [m]	5,548,077.613	0.018	0.005
GS18T	Y [m]	7,422,240.049	−0.024	0.015
	H [m]	204.005	0.031	0.020
	X [m]	5,548,077.616	0.015	0.018
ZED-F9P + AS10	Y [m]	7,422,240.051	−0.026	0.009
	H [m]	204.028	0.009	0.032
	X [m]	5,548,077.618	0.013	0.011
ZED-F9P + ANN-MB-00	Y [m]	7,422,240.051	−0.026	0.017
	H [m]	204.002	0.035	0.042

**Table 7:** Measurement results on Pt 6.

Receiver		Coordinate	Ref. Difference	St. Deviation
	X [m]	5,548,031.736	0.004	0.021
GS18T	Y [m]	7,422,213.194	−0.008	0.025
	H [m]	204.576	−0.004	0.038
	X [m]	5,548,031.743	−0.004	0.011
ZED-F9P + AS10	Y [m]	7,422,213.195	−0.009	0.016
	H [m]	204.582	−0.010	0.031
	X [m]	5,548,031.742	−0.002	0.018
ZED-F9P + ANN-MB-00	Y [m]	7,422,213.196	−0.010	0.022
	H [m]	204.563	0.009	0.031

**Table 8:** Measurement results on Pt 7.

Receiver		Coordinate	Ref. Difference	St. Deviation
	X [m]	5,548,075.284	−0.004	0.029
GS18T	Y [m]	7,422,190.919	0.001	0.047
	H [m]	205.945	−0.032	0.057
	X [m]	5,548,075.344	−0.064	0.075
ZED-F9P + AS10	Y [m]	7,422,190.954	−0.033	0.106
	H [m]	205.875	0.038	0.015
	X [m]	5,548,075.259	0.021	0.110
ZED-F9P + ANN-MB-00	Y [m]	7,422,190.852	0.069	0.147
	H [m]	205.676	0.237	0.392

## Data Availability

The source data can be sent after an e-mail inquiry.

## Abbreviations

BDS	BeiDou Navigation Satellite System
CEP	Circular Error Probable
GAL	Galileo Navigation Satellite System
GLO	GLONASS—GLObal’naya NAvigatsionnaya Sputnikovaya Sistema
GNSS	Global Navigation Satellite System
GPS	Global Positioning System
Hz	horizontal or hertz (unit of frequency), depending on meaning
IPxx	International Protection Rating
L1C/A	L2C, L1OF, L2OF, E1B/C, E5b, B1l, B2l—GNSS signals
MSD	Mean Standard Deviation
PCO	Phase Center Offset
PCV	Phase Center Variation
ppm	part per million
RMSE	Root Mean Square Error
RTCM	Radio Technical Commission for Maritime
RTK	Real Time Kinematic
RTN	Real Time Kinematic Network
V	vertical
